# Editorial: Protein lipidation in health and disease

**DOI:** 10.3389/fphys.2023.1317031

**Published:** 2023-10-30

**Authors:** William Fuller, Rebeca Mejías

**Affiliations:** ^1^ Institute of Cardiovascular and Medical Sciences, University of Glasgow, Glasgow, United Kingdom; ^2^ Department of Physiology, School of Biology, Universidad de Sevilla, Seville, Spain; ^3^ Instituto de Biomedicina de Sevilla-IBiS, Hospital Universitario Virgen Del Rocío, Consejo Superior de Investigaciones Científicas, Universidad de Sevilla, Seville, Spain

**Keywords:** palmitoylation, lipidation, post-translational modifications, human pathologies, palmitoyl acyltransferase (PAT)

Protein lipidation, the attachment of lipids to proteins, influences how proteins fold, their stability, trafficking, interaction with other proteins, and association with specific domains on cellular membranes. Various types of lipids, such as phospholipids, isoprenoids, fatty acids, cholesterol, GPI (glycosylphosphatidylinositol)-anchors, and LDEs (lipid-derived electrophiles), modify proteins covalently. S-palmitoylation or S-acylation is a reversible type of lipidation where a fatty acid called palmitate attaches to specific cysteine residues through a labile thioester bond. N-palmitoylation (via a stable amine bond) and O-palmitoylation (via a stable ester bond) are less common and irreversible kinds of palmitoylation (Chen et al., 2018). S-palmitoylation (called palmitoylation for simplicity in this editorial) is catalysed in mammals by a family of 23–24 proteins called palmitoyl acyltransferases (zDHHC-PATs), and de-acylation is mediated by α/β serine hydrolases and thioesterases, including APT1 (acyl-protein thiosterase 1) and ABHD17 thioesterases, among others (Zhou et al., 2023). Additionally, several PAT-interacting proteins have been identified that regulate their function and localization (Salaun et al., 2020).

The objective of this Research Topic is to compile the most recent insights on the impact of protein lipidation on protein function and explore how alterations in these modifications are linked to the pathophysiology of human diseases, paving the way for the development of novel therapeutic strategies.

One important feature of many post-translational modifications (PTMs) is how they can be regulated by gene-splicing variants. For example, the splice variant of the AMPA scaffolding protein GRIP1, GRIP1b, can be palmitoylated, whereas GRIP1a cannot, affecting its subcellular distribution (Hanley and Henley, 2010). In this line, Wirth and Ponimaskin review the literature describing that the small GTPase Cdc42 splice variant which is palmitoylated (Cdc42-palm) is mainly expressed in the brain, in contrast to its prenylated ubiquitous form (Cdc42-prenyl). It would be interesting to further explore in the future whether these are anecdotal cases or whether the regulation of splice variants and protein isoforms are more widespread modulators of protein palmitoylation.

Recent studies have revealed that numerous immune-associated proteins undergo S-palmitoylation. These findings have shed light on the vital roles played by palmitoylation in immune function, particularly in directing immune signalling proteins to the membrane and lipid rafts. Furthermore, alterations in PAT activity and variations in palmitoylation have been linked to human immunological disorders (Zhang et al., 2021). To strengthen the functional importance of lipidation in the immune system, Wirth and Ponimaskin discuss the possible association between aberrant palmitoylation of Cdc42 and activation of inflammatory responses and autoinflammatory diseases. Furthermore, West et al. introduce PATs as novel regulators of T-cell function in their literature review. For example, zDHHC7 and zDHHC21 have been shown to be critical for T-helper cells differentiation, and key proteins in T-cell signalling such as CD4 and CD8 are palmitoylated. Despite these advances, the role of lipidation in the immune system remains mostly unknown.

Diverse findings point to a complex interrelationship of palmitoylation and other PTMs such as phosphorylation, S-nitrosylation, acetylation and ubiquitination. Thus, for example, palmitoylation may inhibit, compete with, or facilitate another PTM, and palmitoylation/depalmitoylation can also regulate subsequent phosphorylation of certain substrates [reviewed by (Ramzan et al.)]. In the future it will be pivotal to further investigate the crosstalk between lipidation and other PTMs to understand the consequences for protein localization and function, as well as the impact on organelle function.

A fundamental limitation in the lipidation field, is the limited availability of assays to assess palmitoylation. Schek et al. describe a methodological advance for detecting palmitoylated proteins on paraffin-embedded samples. This approach could provide important insights into the role of palmitoylation in the pathology of human diseases and be extended to detect additional PTMs. An additional drawback has been the relatively unexplored pattern of expression of different PATs and their interacting/regulatory proteins. To address this, Wild et al. have generated databases of the expression of palmitoylation and depalmitoylation enzymes, and accessory proteins, in mouse brain (BrainPalmSeq) (Wild et al., 2022) and human cell types (CellPalmSeq). These tools will help both clinicians and researchers to interrogate the implications of palmitoylation in physiology and pathology. CellPalmSeq, described in this Research Topic, is an important resource that will support the field to select the most appropriate cellular models to interrogate how palmitoylation regulates pathways and individual proteins.

Dysregulation of lipid metabolism and protein lipidation is associated with different pathologies, but understanding the underlying molecular mechanisms remains a challenge. This is partly due to limited knowledge regarding how lipidation and its regulating enzymes are controlled by cellular signalling pathways. While lipidation-targeted therapies are being investigated for cancer treatment, for example, their application to other diseases is still largely unexplored. Furthermore, while certain forms of protein lipidation, like S-palmitoylation, have garnered substantial attention in recent years, others, such as N-palmitoylation, remain relatively unexplored. Therefore, it is crucial to advance the knowledge of the regulatory mechanisms that control lipidation, the interplay between lipidation and other post-translational modifications, methodologies to detect, measure, and manipulate protein lipidation, the interplay between lipid metabolism and protein lipidation, and less common or studied varieties of lipidation. Besides, it is worth to highlight the importance of prioritising studies investigating putative sex difference in palmitoylation (Ramzan et al.) and palmitoylation-independent functions of PATs in future studies.

The contribution of aberrant palmitoylation to development of human pathologies is an important consideration for this field. This Research Topic gives a broad overview of the importance of these PTMs in neurodegeneration, X-linked intellectual disability, metabolic disease, cancer, and cardiac pathologies (Lemarié et al.; Main et al.; Nguyen et al.; Pierce and Hougland; Ramzan et al.). Main et al. used publicly-available RNA-seq datasets from patients with heart failure to evaluate how palmitoylation and depalmitoylation pathways remodel in these patients as well as in animal models and clinical biopsies. A complex picture emerges in which the surface-membrane acyl transferase zDHHC5 expression increases significantly during early remodeling but declines once heart failure is established. These expression changes correlate very poorly with substrate palmitoylation, highlighting the continuing paucity in our understanding of how zDHHC-PAT activity is controlled.

With this in mind, the findings of Nguyen et al. that several zDHHC-PATs are stabilised by accessory proteins, is important for the field. Although zDHHC20, the first zDHHC enzyme to be crystallized is clearly functional without such a partner, Nguyen et al. find that zDHHCs 5, 8, 9, 14, and 18 (but not 3 and 20) can be stabilized by GCP16 to support enhanced enzymatic activity. This suggests an additional layer of control for activity of some of these enzymes than has hitherto been under-appreciated. Clearly the expression level of the accessory protein, as well as the expression level of the enzyme itself, will be an important determinant of substrate palmitoylation.

Numerous ion transporters and channels in excitable tissues are regulated by palmitoylation. Congreve et al. focused on the cardiac voltage sensitive ion channel HCN4, which is expressed in the sino atrial node and cardiac conduction system, and has the unique property of being activated by hyperpolarization. HCN4 controls heart rate by controlling the rate of depolarization in nodal tissue, and its activity is significantly enhanced by palmitoylation of two cysteines in the channel’s amino terminus. This raises the intriguing possibility that a fundamental feature of multicellular organisms, the heartbeat, is controlled by the coordinated activities of palmitoylating and depalmitoylating enzymes on a single ion channel.

The scaffolding protein ankyrin B, the small cousin of the giant ankyrin G, controls the localization of ion channels and transporters in numerous tissues. Gupta and Jenkins identified numerous palmitoylation sites in ankyrin B which are palmitoylated by zDHHC17. When ankyrin B is not palmitoylated it loses its ability to correctly localize voltage sensitive sodium channels to dendritic membranes, highlighting the importance of palmitoylation not only of ion channels but also of their targeting partners for the control of tissue excitability.

In the last 20 years, progress has been made in the study of regulatory mechanisms of palmitoylation, the role of this posttranslational modification in physiology and diseases, and the methodology for its study. However, there is still much research to be performed in these aspects and the interplay of the findings with the overall regulation of lipid metabolism in the organism.
